# Comparative evaluation of fluoride varnishes, self-assembling peptide-based remineralization agent, and enamel matrix protein derivative on artificial enamel remineralization in vitro

**DOI:** 10.1186/s40510-020-00345-1

**Published:** 2021-01-25

**Authors:** Yağmur Lena Sezici, Enver Yetkiner, Arzu Aykut Yetkiner, Ece Eden, Rengin Attin

**Affiliations:** 1 Private Practice, Izmir, Turkey; 2 Department of Orthodontics, Faculty of Dentistry, Ege University, Izmir, Turkey; 3 Department of Pedodontics, Faculty of Dentistry, Ege University, Izmir, Turkey; 4 Clinic for Orthodontics and Pediatric Dentistry, University of Zurich, Zurich, Switzerland

**Keywords:** White spot lesions, Remineralization, Light-induced fluorescence, Fluoride varnish, Enamel matrix protein, Self-assembling peptide

## Abstract

**Background:**

One of the most unfavorable side effects of fixed orthodontic treatment is white spot lesions (WSLs). Although the most important approach is prevention of WSLs, it is also essential to evaluate the efficacy of the remineralization agents. However, there is no concurrence in the literature with respect to the remineralization process of these agents. The objective of the present study was to evaluate the effects of different fluoride varnishes, enamel matrix protein, and self-assembling peptide derivatives with varying chemical compositions on remineralization of artificially created WSLs in vitro using quantitative light-induced fluorescence (QLF).

**Methods:**

Artificial WSLs were created on bovine enamel samples using acidic buffer solution (pH 5, 10 days). Specimens were randomly allocated to six groups (*n* = 10/group): (1) Emdogain (Straumann, Basel, Switzerland), (2) Curodont Repair (Credentis AG, Switzerland), (3) Duraphat (Colgate-Palmolive, New York, NY), (4) Clinpro XT (3 M ESPE, Pymble, New South Wales, Australia), (5) Enamel Pro Varnish (Premier Dental Products, PA, USA), and (6) control (untreated). The agents were applied to the WSLs according to the manufacturers’ instructions. Fluorescence loss (Δ*F*), lesion area (area), and impact (Δ*Q*) values of enamel surfaces were quantified by QLF-D Biluminator^TM^ (Inspektor-Pro, Amsterdam, The Netherlands) at baseline and after 7, 14, and 21 days of application of the respective materials.

**Results:**

Δ*F* value presented a significantly decreasing trend throughout the 21 days for all groups except the Duraphat and Enamel Pro varnishes. The changes between 14th and 21st days of the Clinpro XT varnish application were significantly higher than Emdogain, Curodont, and Enamel Pro. The Curodont group showed higher lesion area changes between the first and second week in comparison to the Emdogain, Clinpro XT, and Enamel Pro groups, whereas Clinpro XT assured the highest reduction from the second to the third week of the observation period.

**Conclusions:**

The fluorescence loss was significantly reduced with enamel matrix protein, self-assembling peptide, and light-curable fluoride varnishes in the analysis for 21 days. Curodont and Clinpro XT were effective in diminishing the fluorescence loss and lesion area compared to the Duraphat, Enamel Pro fluoride varnishes, and Emdogain in different time points.

## Background

White spot lesion (WSL) is the first clinical sign of enamel demineralization which may progress to dental caries or an arrested demineralized area if not treated at an early stage [1]. Fixed orthodontic appliances cause retentive areas that are difficult to ensure the oral hygiene, which leads to plaque accumulation. The risk of WSL formation increases due to orthodontic treatment with a reported incidence of 46–73% [1, 2], especially in patients with poor oral hygiene [3].

The most important aspect of WSL management is the prevention of new lesions forming. Good oral hygiene maintenance, reducing dietary carbohydrate consumption, and using fluoridated dental care materials constitute the main prerequisites for this purpose [4]. For the treatment of already present lesions, minimally invasive treatments are advocated for the maximum preservation of healthy enamel and remineralization of affected areas [4, 5]. Current approaches for remineralization of initial lesions aim to reduce the solubility of enamel by inhibiting the demineralization of hydroxyapatites. The most established element for prevention against caries is fluoride, which aims to harden the mineral surface layer and inhibit its progression. Fluoride, best known for its preventive effect on caries, aims at hardening the mineral surface layer, thus inactivating the caries by inhibiting its progression [5]. Among all the methods of fluoride application, fluoride varnish is the preferred method as they are less dependent on patient compliance [6]. However, fluoride does not diffuse in the demineralized subsurface zone [7] and conventional fluoride varnish have to be applied repeated range from once every 2 weeks to four topical applications a year to maintain their effectiveness. Currently, fluoride varnish application is being used in a conventional and light-curable form to reduce the frequent application [6].

With the advent of enamel matrix proteins, self-assembling peptides were shown to be used in the remineralization process of enamel, dentin, and cementum [6]. Self-assembling peptides form a three-dimensional matrix that helps the remineralization of subsurface lesions. The short hydrophilic peptide within their molecular design enables the assembly of the structure into the fibers. It has been reported that the self-assembling peptide P_11-4_ with high affinity to calcium ions in saliva also leads to the formation of enamel crystals around the enamel matrix. The self-assembling peptide described in combination with guided enamel regeneration is based on the rational design of a short hydrophilic peptide that assembles into fibers, forming a 3-dimensional matrix that has been used for remineralization of subsurface lesions [7, 8]. It has been reported that the self-assembling peptide P_11-4_ with high affinity to calcium ions in saliva also leads to the formation of enamel crystals around the enamel matrix [9]. As a commercially available product, Emdogain, which is mainly used for guided tissue regeneration, is composed of the hydrophobic enamel matrix proteins [10]. This revolutionary product also has attracted much attention for the remineralization process of enamel in the past years. Its protein constituents have been claimed to have the potential to advocate remineralization process of enamel and dentin [10, 11]. However, only a few studies [11–14] have been evaluated its potential.

There is a growing body of literature that recognizes the role of fluoride varnishes; however, to the best of our knowledge, despite the frequent use of fluoride products as prophylaxis, no in vivo or in vitro study has directly or indirectly compared the effect of varying fluoride varnishes, self-assembling peptides, and enamel matrix protein on enamel demineralization. Therefore, the primary objective of this study was to compare the effects of topical application of conventional fluoride varnishes, light-curable fluoride varnish, self-assembling peptides, and enamel matrix proteins in the treatment of enamel demineralization in vitro by means of quantitative light-induced fluorescence (QLF). The null hypothesis was that the application of fluoride varnishes, self-assembling peptides, and enamel matrix proteins cannot improve the remineralization process of WSLs.

## Methods

### Sample preparation

Bovine incisors (*n* = 10/group) stored in 0.5% chloramine solution at 4 °C no longer than 6 months were initially cut from their roots under water irrigation. Enamel discs with a diameter of 5 mm were cut from the labial aspect of each crown using a custom-made diamond-coated trephine bur (80 μm, Intensiv SA, Lugano-Grancia, Switzerland). They were then flattened from the bottom to approximately 3 mm in height (Struers, Birmensdorf, Switzerland). Each piece was randomly allocated to six groups. They were embedded with their labial surfaces exposed in auto-polymerizing acrylic resin (Palapress; Heraeus Kulzer, Wehrheim, Germany) in cylindrical molds with a size of 6 mm diameter and 3 mm thickness. The specimens were ground flat and polished with water-cooled carborundum discs (1200, 2400, and 4000 grit; Struers, Erkrat, Germany) and stored in tap water until demineralization procedure. The enamel surface was covered with a perforated tape, leaving a defined area in 3 mm diameter. The remaining area was demineralized in an acidic buffer solution for 14 days.

### Demineralization procedure

**Fig. 1:**
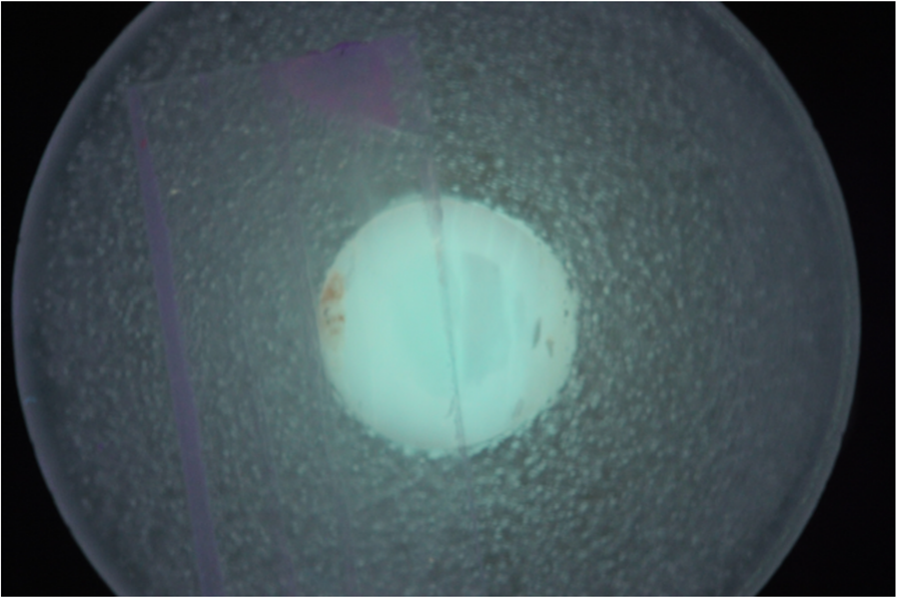
The opacity change of a demineralized bovine incisor

Demineralization was achieved by immersing the specimens in acidic buffer solution (pH 5, 37 °C, 10 days) according to Buskes et al. [15]. The solution was renewed each second day to keep the pH constant. Subsequently, specimens were removed from the solution and thoroughly washed with deionized water. Examination of samples was performed under magnification to determine the opacity change before the experiment (Fig. 1).

### Remineralization procedures

Following demineralization, the specimens were treated by one-time application of the undermentioned groups as follows:

1. Enamel matrix proteins: Lyophilized Emdogain (Straumann, Basel, Switzerland) was dissolved in 1 mL 0.05 M acetic acid and stored for 30 min at 4 °C to a final concentration of 30 mg/mL and delivered from syringes loaded with 0.7 mL. During each treatment, one drop of Emdogain gel was placed on the surface of the carious lesion and daubed into a very thin layer that covered the lesion surface.
2. Self-assembling peptide (SAP): Curodont (Curodont Repair, Credentis AG, Switzerland) (P_11-4_ oligopeptide solution) was mixed with 0.05 mL deionized water, and one drop was applied and left for 5 min on the demineralized enamel surfaces.
3. Duraphat varnish (Colgate-Palmolive, New York, NY) (5% NaF and 2.26% F): The enamel surface received a single topical application of a conventional fluoride varnish with a brush applicator after mixing thoroughly.
4. Clinpro XT (3 M ESPE, Pymble, New South Wales, Australia): The paste and the liquid components were mixed for 15 s using an agate spatula. Consistent mix was then applied as a thin layer over the etched enamel surface with the aid of applicator brush and light-cured for 20 s.
5. Enamel Pro Varnish (Premier Dental Products, PA, USA): Varnish was stirred with an applicator brush and mixed thoroughly. A thin coat of varnish applied across tooth surfaces.
6. Control: Specimens remained untreated. The chemical compositions of the materials are summarized in Table 1. All procedures involving air/water syringe were performed using a custom-made device assuring standard distance to the specimens from the application tips and standard pressures provided by the air/water syringe. After storing the specimens in artificial saliva at room temperature for 4 h, the remineralization products were carefully removed with the swabs and 50% acetone solution. The tapes were then removed and the specimens were then submerged back into the renewed artificial saliva to imitate the intraoral conditions. Artificial saliva was prepared according to the formula given by Klimek et al. [16] and was renewed every 2 days. The pH value of the artificial saliva solution for the groups was measured ranged between 6.5 and 6.8.

**Table 1:** Composition of the products according to the manufacturers’ information

Product	Manufacturer	Chemical composition
Emdogain	Straumann, Basel, Switzerland	Composed of a mixture of hydrophobic enamel matrix proteins derived from 6-month-old porcine tooth buds containing amelogenin, enamelin, tuftelin, amelin, and ameloblastin, in a propylene glycol alginate (PGA).
Curodont	Curodont Repair, Credentis AG, Switzerland	P_11-4_ peptide
Duraphat	Colgate-Palmolive, New York, NY	5% sodium fluoride varnish (22,600 ppm fluoride)
Clinpro	3 M ESPE, Pymble, New South Wales, Australia	5% sodium fluoride white varnish, tri-calcium phosphate
Enamel Pro	Premier Dental Products, PA, USA	5% sodium fluoride varnish, ACP formula

### QLF measurements

At baseline (after the demineralization process) (T0) and after 7 (T1), 14 (T2), and 21 (T3) days, QLF images were taken by the same author (A.A.Y) using QLF-D Biluminator^TM^ (Inspektor Research Systems BV, Amsterdam, The Netherlands). Between three and five views were taken of the specimens so that good quality images could be captured of each lesion. Each sample was dried for 5 s with compressed air before imaging. Images were captured using a custom-built, high-resolution, fluorescence imaging system. The camera and illuminator were mounted in a geometry stabilizing unit (State-of-the-art In Vitro Research setup). This, together with video repositioning software, enabled specimens to be accurately repositioned at each observation. The images were acquired using the QLF-D Biluminator^TM^ software (Inspektor Research Systems BV, Amsterdam, The Netherlands) with the video repositioning software and a grab level of 0.95 set for the 7-, 14-, and 21-day images. The obtained images were stocked on a hard drive and a unique subject identifier was allocated.

QLF provides mainly 3 variables: Δ*F* (%), area (mm^2^), and Δ*Q* (mm^2^ × %). Δ*F* is the percentage fluorescence loss comparing sound enamel to an identified lesion. Threshold for Δ*F* is set to 5%, indicating that any fluorescence loss below this level is considered as noise. Area of the lesion is calculated as pixels considered by the analysis software as representing demineralized enamel (i.e., those with a fluorescence loss of 5% or more). Δ*Q* is the product of Δ*F* and area and indicates the volume of the lesion [17]. After the calculation of the samples’ Δ*F*, Δ*F* max, area, and Δ*Q* values, the data were exported as text files and imported into Excel (Microsoft, Redmond, WA) using a custom macro.

### Statistical analysis

A sample size of 10 in each group was calculated to have 80% power to detect a difference in means of 1.69 according to our pilot study results. This assumes that the group 1 standard deviation is 6.72 and the group 2 standard deviation is 7.35 using a two-group Satterthwaite *t* test with a 0.05 two-sided significance level.

Statistical analysis was performed with SPSS software, version 21.0 (SPSS Inc., Chicago, IL, USA). A Shapiro-Wilk test was performed to assess the distribution of data. As the data were normally distributed, one-way ANOVA was conducted to analyze possible differences between the groups at the same time points and the differences between the time points. The mean difference in Δ*F*, Δ*F* max, area, and Δ*Q*, observed in different time points within each group, was analyzed using one-way ANOVA, followed by Tukey’s post hoc test. Statistical significance was established at *p* < 0.05.

## Results

The mean Δ*F*, Δ*F* max, area, and Δ*Q*, standard deviation, and minimum/maximum values of measurements at baseline, after 7, 14, and 21 days for each group are presented in Table 2. The baseline parameters (Δ*F*, Δ*F* max, area, and Δ*Q*) did not differ significantly among the 6 groups (*p* = 0.793, *p* = 0.538, *p* = 0.449, and *p* = 0.108, respectively). The changes obtained between time points and significant differences between groups at each time point are shown in Table 3.

**Table 2:** Intragroup comparisons of QLF scores between timeline intervals

Groups	Time intervals	Output	Minimum	Maximum	Mean	SD	***P*** (∆***F***)	***P*** (∆***F*** max)	***P*** (∆***Q***)	***P*** (area)
**Control**	**T0**	∆*F*_0_	14.30	34.50	26.03 A	8.12	0.001	0.007	0.001	0.016
		∆*F*_0_ max	27.00	55.00	40.13 A	9.34				
		∆*Q*_0_	53,670.00	213,802.00	110,111.88 A	51,720.45				
		Area_0_	2017.00	6419.00	4263.50 A	1384.97				
	**T1**	∆*F*_1_	6.30	29.40	17.70 A	7.11				
		∆*F*_1_ max	10.00	40.00	28.38 A	10.68				
		∆*Q*_1_	1220.00	112,055.00	62,244.00 A	39,148.39				
		∆Area_1_	195.00	5028.00	3184.00 A	1551.10				
	**T2**	∆*F*_2_	0.00	23.20	13.71 B	8.93				
		∆*F*_2_ max	0.00	45.00	24.25 A	16.10				
		∆*Q*_2_	0.00	84,561.00	46,730.75 B	33,183.38				
		∆Area_2_	0.00	5505.00	2612.13 A	1930.73				
	**T3**	∆*F*_3_	0.00	19.00	8.36 B	7.68				
		∆*F*_3_ max	0.00	32.00	15.50 B	14.36				
		∆Q_3_	0.00	64,173.00	22,160.13 B	25,505.00				
		∆Area_3_	0.00	3436.00	1464.63 B	1599.99				
**Emdogain**	**T0**	∆*F*_0_	11.20	24.40	20.00 A	4.64	0.001	0.027	0.005	0.216
		∆*F*_0_ max	25.00	36.00	32.38 A	3.66				
		∆*Q*_0_	1394.00	137,731.00	89,036.00 A	41,904.33				
		Area_0_	124.00	6798.00	4290.13 A	2095.13				
	**T1**	∆*F*_1_	8.30	15.80	10.66 B	2.53				
		∆*F*_1_ max	15.00	31.00	20.50 B	5.53				
		∆*Q*_1_	2503.00	70,911.00	31,330.75 B	22,947.29				
		∆Area_1_	192.00	5262.00	2883.13 B	1700.05				
	**T2**	∆*F*_2_	7.40	22.10	13.23 B	5.78				
		∆*F*_2_ max	13.00	38.00	24.75 A	9.57				
		∆*Q*_2_	11,680.00	110,764.00	49,656.13 A	41,037.88				
		∆Area_2_	1568.00	5828.00	3288.63 A	1685.34				
	**T3**	∆*F*_3_	6.10	16.70	10.89 B	3.86				
		∆*F*_3_ max	9.00	41.00	21.88 A	10.87				
		∆*Q*_3_	2253.00	71,179.00	28,932.88 A	25,220.13				
		∆Area_3_	226.00	4274.00	2405.88 A	1693.33				
**Curodont**	**T0**	∆*F*_0_	13.60	36.40	22.93 A	7.63	< 0.001	< 0.001	< 0.001	< 0.001
		∆*F*_0_ max	28.00	55.00	38.25 A	8.96				
		∆*Q*_0_	74,907.00	205,354.00	115,478.38 A	52,158.27				
		Area_0_	3907.00	6734.00	5414.38 A	1000.16				
	**T1**	∆*F*_1_	8.30	27.50	18.40 A	7.43				
		∆*F*_1_ max	16.00	43.00	30.88 A	9.20				
		∆*Q*_1_	17,690.00	163,146.00	88,866.75 A	50,511.48				
		∆Area_1_	2140.00	6054.00	4597.50 A	1286.11				
	**T2**	∆*F*_2_	5.60	17.00	10.03 BB	4.45				
		∆*F*_2_ max	7.00	29.00	16.58 BB	9.31				
		∆*Q*_2_	67.00	91,812.00	26,772.50 BB	33,943.70				
		∆Area_2_	12.00	5403.00	2174.13 BB	1844.96				
	**T3**	∆*F*_3_	5.50	15.30	8.54 BB	3.46				
		∆*F*_3_ max	6.00	29.00	16.75 BB	8.33				
		∆*Q*_3_	90.00	53,863.00	17,041.88 BB	20,199.98				
		∆Area_3_	16.00	3527.00	1748.75 BB	1326.62				
**Duraphat**	**T0**	∆*F*_0_	6.90	42.30	20.00 A	14.95	0.313	0.641	0.348	0.405
		∆*F*_0_ max	11.00	61.00	32.00 A	20.14				
		∆*Q*_0_	5531.00	282,872.00	95,174.13 A	117,752.60				
		Area_0_	749.00	6681.00	3475.25 A	2186.93				
	**T1**	∆*F*_1_	7.00	37.30	16.45 A	13.09				
		∆*F*_1_ max	13.00	60.00	28.88 A	19.25				
		∆*Q*_1_	8228.00	228,229.00	69,704.63 A	85,594.07				
		∆Area_1_	1177.00	6332.00	3053.50 A	1923.82				
	**T2**	∆*F*_2_	6.90	29.00	14.16 A	9.27				
		∆*F*_2_ max	12.00	55.00	25.13 A	16.69				
		∆*Q*_2_	1189.00	141,413.00	42,272.00 A	47,123.66				
		∆Area_2_	172.00	4877.00	2360.25 A	1535.93				
	**T3**	∆*F*_3_	0.00	23.30	9.23 A	6.62				
		∆*F*_3_ max	0.00	48.00	20.75 A	16.52				
		∆*Q*_3_	0.00	124,739.00	27,494.13 A	41,258.29				
		∆Area_3_	0.00	5363.00	1974.13 A	1918.22				
**Clinpro XT**	**T0**	∆*F*_0_	7.70	34.10	21.24 A	10.41	0.002	0.001	0.012	< 0.001
		∆*F*_0_ max	13.00	48.00	33.50 A	11.95				
		∆*Q*_0_	20,036.00	230,424.00	101,547.25 A	74,454.11				
		Area_0_	2587.00	6749.00	4223.13 A	1438.53				
	**T1**	∆*F*_1_	6.20	30.80	15.04 A	7.70				
		∆*F*_1_ max	10.00	52.00	29.25 A	14.93				
		∆*Q*_1_	1255.00	210,619.00	58,227.5 A	66,253.18				
		∆Area_1_	167.00	6831.00	2975.63 A	2070.24				
	**T2**	∆*F*_2_	9.00	23.60	17.33 A	4.93				
		∆*F*_2_ max	19.00	41.00	30.13 A	8.56				
		∆*Q*_2_	12,163.00	158,883.00	71,085.00 A	44,661.17				
		∆Area_2_	1354.00	6745.00	3785.75 A	1585.04				
	**T3**	∆*F*_3_	0.00	8.00	6.16 BB	2.62				
		∆*F*_3_ max	0.00	17.00	10.63 BBB	5.21				
		∆*Q*_3_	0.00	10,941.00	4806.00 B	5189.88				
		∆Area_3_	0.00	1390.00	631.75 BBB	651.32				
**Enamel Pro**	**T0**	∆*F*_0_	11.50	38.20	20.79 A	8.19	0.569	0.159	0.450	0.370
		∆*F*_0_ max	19.00	53.00	31.88 A	10.60				
		∆*Q*_0_	39,319.00	197,340.00	88,195.13 A	51,282.32				
		Area_0_	3004.00	51,645.00	9850.13 A	16,900.88				
	**T1**	∆*F*_1_	6.40	35.50	16.74 A	10.28				
		∆*F*_1_ max	9.00	57.00	27.00 A	16.07				
		∆Q_1_	1715.00	138,231.00	57,311.88 A	50,640.73				
		∆Area_1_	269.00	4555.00	2766.63 A	1463.68				
	**T2**	∆*F*_2_	5.60	29.70	16.10 A	8.59				
		∆*F*_2_ max	6.00	45.00	24.25 A	13.83				
		∆*Q*_2_	6.00	140,987.00	59,021.38 A	55,600.69				
		∆Area_2_	1.00	9384.00	3720.25 A	3017.98				
	**T3**	∆*F*_3_	5.40	30.10	14.70 A	8.53				
		∆*F*_3_ max	5.80	33.00	17.29 A	8.73				
		∆*Q*_3_	5.00	120,394.00	49,563.70 A	43,089.68				
		∆Area_3_	1.00	8697.00	4039.13 A	2642.31				

*SD* indicates standard deviation. Comparisons of the measurements for each treatment at different time points that are not significantly different are marked with the same uppercase letters

**Table 3:** Intergroup comparisons of the QLF measurements’ changes between different time points

Time periods	Group	Output	Minimum	Maximum	Mean	SD	***P*** (∆***F***)	***P*** (∆***F*** max)	***P*** (∆***Q***)	***P*** (area)
**T1-T0**	**Control**	∆*F*	− 17.00	− 1.10	− 8.33 A	5.69	0.207	0.205	0.348	0.386
		∆*F* max	− 22.00	− 2.00	− 11.75 A	7.34				
		∆*Q*	− 101,747.00	− 13,521.00	− 47,867.88 A	37,369.17				
		Area	− 4157.00	1250.00	− 1079.50 A	1588.22				
	**Emdogain**	∆*F*	− 14.60	1.80	− 9.34 A	5.30				
		∆*F* max	− 18.00	6.00	− 11.88 A	7.83				
		∆*Q*	− 86,808.00	1109.00	− 57,705.25 A	29,299.65				
		Area	− 5064.00	68.00	− 1407.00 A	1600.74				
	**Curodont**	∆*F*	− 9.50	− 1.60	− 4.53 A	2.59				
		∆*F* max	− 14.00	− 2.00	− 7.38 A	4.07				
		∆*Q*	− 57,217.00	− 10,041.00	− 26,611.63 A	16,529.55				
		Area	− 3349.00	405.00	− 816.88 A	1159.37				
	**Duraphat**	∆*F*	− 16.50	7.90	− 3.55 A	7.51				
		∆*F* max	− 21.00	18.00	− 3.13 A	12.76				
		∆*Q*	− 109,326.00	47,995.00	− 25,469.50 A	50,026.95				
		Area	− 2254.00	1468.00	− 421.75 A	1415.32				
	**Clinpro XT**	∆*F*	− 15.70	− 0.10	− 6.20 A	6.09				
		∆*F* max	− 23.00	10.00	− 4.25 A	9.95				
		∆*Q*	− 104,528.00	− 9011.00	− 43,319.75 A	38,896.37				
		Area	− 2450.00	82.00	− 1247.50 A	900.27				
	**Enamel Pro**	∆*F*	− 12.70	0.00	− 4.05 A	4.56				
		∆*F* max	− 18.00	4.00	− 4.88 A	8.41				
		∆*Q*	− 59,109.00	− 1659.00	− 30,883.25 A	22,864.84				
		Area	− 47,753.00	− 45.00	− 7083.50 A	16,470.76				
**T2-T1**	**Control**	∆*F*	− 11.30	4.30	− 3.99 A	4.98	0.001	0.001	< 0.001	0.001
		∆*F* max	− 18.00	5.00	− 4.13 A	7.22				
		∆*Q*	− 47,304.00	12,798.00	− 15,513.25 A	20,137.28				
		Area	− 4184.00	477.00	− 571.88 A	1496.28				
	**Emdogain**	∆*F*	− 7.30	12.30	2.56 A	7.00				
		∆*F* max	− 12.00	19.00	4.25 A	9.68				
		∆*Q*	− 48,086.00	76,082.00	18,325.38 A	42,654.17				
		Area	− 1797.00	2886.00	405.50 A	1723.90				
	**Curodont**	∆*F*	− 14.40	0.90	− 8.38 B	4.78				
		∆F max	− 24.40	1.00	− 14.30 B	8.42				
		∆*Q*	− 97,885.00	6214.00	− 62,094.25 B	30,329.38				
		Area	− 4415.00	453.00	− 2423.38 B	1710.05				
	**Duraphat**	∆*F*	− 10.60	0.70	− 2.29 A	4.15				
		∆*F* max	− 14.00	1.00	− 3.75 A	4.68				
		∆*Q*	− 105,347.00	8796.00	− 27,432.63 A	43,319.05				
		Area	− 2094.00	1039.00	− 693.25 A	946.19				
	**Clinpro XT**	∆*F*	− 7.20	9.20	2.29 A	5.73				
		∆*F* max	− 12.00	13.00	0.88 AC	10.55				
		∆*Q*	− 51,736.00	59,677.00	12,857.50 AC	35,486.55				
		Area	− 1040.00	2812.00	810.13 AC	1284.18				
	**Enamel Pro**	∆*F*	− 6.10	5.60	− 0.64 A	4.00				
		∆*F* max	− 12.00	4.00	− 2.75 A	5.47				
		∆*Q*	− 35,100.00	25,841.00	1709.50 AC	19,359.69				
		Area	− 1507.00	6214.00	953.63 AC	2285.35				
**T3-T2**	**Control**	∆*F*	− 16.40	3.10	− 5.35 A	6.37	0.007	0.021	0.010	0.003
		∆*F* max	− 26.00	5.00	− 8.75 A	11.04				
		∆*Q*	− 84,039.00	9708.00	− 24,570.63 A	29,727.75				
		Area	− 5428.00	0.00	− 1147.50 A	1890.12				
	**Emdogain**	∆*F*	− 8.60	8.20	− 2.34 A	6.28				
		∆*F* max	− 11.00	26.00	− 2.88 A	12.26				
		∆*Q*	− 64,486.00	48,354.00	− 20,723.25 A	37,101.79				
		Area	− 2852.00	1594.00	− 882.75 A	1534.34				
	**Curodont**	∆*F*	− 7.00	9.70	− 1.49 A	5.06				
		∆*F* max	− 11.00	21.00	0.18 A	10.23				
		∆*Q*	− 56,413.00	53,796.00	− 9730.63 A	32,246.90				
		Area	− 2577.00	3507.00	− 425.38 A	1849.69				
	**Duraphat**	∆*F*	− 15.80	1.10	− 4.94 A	5.53				
		∆*F* max	− 18.00	28.00	− 4.38 A	14.74				
		∆*Q*	− 56,593.00	14,038.00	− 14,777.88 AC	21,291.83				
		Area	− 1648.00	1316.00	− 386.13 AC	1032.35				
	**Clinpro XT**	∆*F*	− 17.90	− 1.30	− 11.16 B	6.13				
		∆*F* max	− 34.00	− 2.00	− 19.50 B	11.03				
		∆*Q*	− 158,393.00	− 1495.00	− 66,279.00 B	48,597.85				
		Area	− 6660.00	36.00	− 3154.00 B	2081.90				
	**Enamel Pro**	∆*F*	− 4.90	0.70	− 1.40 AC	1.89				
		∆*F* max	− 14.70	3.80	− 6.96 A	6.02				
		∆*Q*	− 44,949.00	1899.00	− 9457.68 AC	16,258.16				
		Area	− 687.00	2343.00	318.88 AC	986.34				
**T3-T0**	**Control**	∆*F*	− 26.50	− 10.70	− 17.66 A	4.91	0.053	0.120	0.371	0.847
		∆*F* max	− 34.00	− 11.00	− 24.63 A	8.81				
		∆*Q*	− 213,280.00	− 3485.00	− 87,951.75 A	60,618.08				
		Area	− 6342.00	1358.00	− 2798.88 A	2607.69				
	**Emdogain**	∆*F*	− 14.50	3.10	− 9.1125 A	5.80429				
		∆*F* max	− 25.00	7.00	− 10.5000 A	11.52637				
		∆*Q*	− 98,877.00	1832.00	− 60,103.1250 A	34,929.37284				
		Area	− 4277.00	428.00	− 1884.2500 A	1812.62318				
	**Curodont**	∆*F*	− 26.40	− 5.80	− 14.39 A	6.94				
		∆*F* max	− 37.00	− 5.00	− 21.50 A	10.94				
		∆*Q*	− 169,955.00	− 28,410.00	− 98,436.50 A	46,423.11				
		Area	− 5503.00	− 388.00	− 3665.63 A	1822.66				
	**Duraphat**	∆*F*	− 31.40	1.40	− 10.78 A	11.42				
		∆*F* max	− 39.00	30.00	− 11.25 A	21.42				
		∆*Q*	− 271,266.00	25,531.00	− 67,680.00 A	100,288.28				
		Area	− 5620.00	2783.00	− 1501.13 A	2627.37				
	**Clinpro XT**	∆*F*	− 28.30	0.30	− 15.08 A	11.07				
		∆*F* max	− 39.00	0.00	− 22.88 A	13.30				
		∆*Q*	− 229,934.00	− 9095.00	− 96,741.25 A	78,618.02				
		Area	− 6664.00	− 1224.00	− 3591.38 A	1993.18				
	**Enamel Pro**	∆*F*	− 10.20	− 2.20	− 6.09 A	2.83				
		∆*F* max	− 20.20	− 9.20	− 14.59 A	4.36				
		∆*Q*	− 78,388.00	5097.00	− 38,631.43 A	29,962.50				
		Area	− 45,677.00	3739.00	− 5811.00 A	16,237.03				

*SD* indicates standard deviation. Comparisons of the measurements for each group at different time intervals that are not significantly different are marked with the same uppercase letters

### Fluorescence loss (Δ*F*)

The application of the Emdogain resulted in a significant decrease of the fluorescence loss during the first week (*p* = 0.001). Following the 14 days, Δ*F* decreased significantly in Emdogain, Curodont, and control groups (*p* = 0.021, *p* = 0.001, *p* = 0.022, respectively). Δ*F* value presented a significantly decreasing trend throughout the 21 days for all groups except the Duraphat and Enamel Pro varnishes. Fluorescence loss was reduced significantly for Curodont than Emdogain and Clinpro XT between 7th and 14th day (*p* = 0.002). The changes between T2 and T3 days of the Clinpro XT varnish application were significantly higher than Emdogain, Curodont, and Enamel Pro.

### Maximum fluorescence loss (Δ*F* max)

Following the application of the products, Δ*F* max value presented a significant decrease in all groups after 21 days except for the Duraphat and Enamel Pro varnishes groups. Highest change obtained between the first and the second week was in the Curodont group. However, the Clinpro XT varnish application was shown a significantly higher reduction than Curodont from the second week to the 21st day.

### Lesion area (area)

A distinct decrease in the lesion area was determined in the control, Curodont, and Clinpro XT groups at the end of the observation period (*p* = 0.009, *p* < 0.001, *p* < 0.001, respectively). The Curodont application induced a significant change in area component even after the 14 days (*p* < 0.001). The Curodont group showed higher lesion area changes between T1 and T2 in comparison to the Emdogain, Clinpro XT, and Enamel Pro® groups, whereas Clinpro XT assured the highest reduction from the second to the third week of the observation period.

### Lesion volume (Δ*Q*)

Although changes of the Δ*Q* value at the end of the whole observation process did not differ significantly between treatment groups (*p* = 0.371), the reduction in ΔQ value was found to be significant in the control (*p* = 0.001), Curodont (*p* < 0.001), and Clinpro XT (*p* = 0.007) groups. The mean lesion volume in the Emdogain group was significantly decreased to the 31,330.75 after the first 7 days (*p* = 0.010). A significant decrease was seen after the second week, and these results were sustained throughout 21 days for the Curodont group. The higher lesion volume changes between T1 and T2 were determined in the Curodont group than Emdogain, Clinpro XT, and Enamel Pro groups (*p* < 0.001, *p* = 0.001, *p* = 0.005, respectively). For the time duration between T2 and T3, the greatest amount of the decrease was seen in the Clinpro XT group. Among the treatment groups, the least affected group for the ΔQ measurement was Duraphat varnish.

## Discussion

Since one of the most undesirable side effects of fixed orthodontic treatment is WSLs, it is important to know the proper treatment approaches and the efficacy of the remineralization agents. However, there is no consensus in the literature with respect to their remineralization capacity. Therefore, the aim of the current study was to investigate the efficacy of enamel matrix protein, self-assembling peptide, and fluoride varnishes for the remineralization of WSLs by using an in vitro model. The effects of the materials were determined as the change in the area of the lesions and total lesion fluorescence. Based on the obtained results, the null hypothesis was rejected since differences in the remineralization capacities were observed among the different materials tested. Self-assembling peptide (Curodont) and light-curable fluoride varnish (Clinpro XT) performed better in diminishing the fluorescence loss and lesion area compared to the remained fluoride varnishes and enamel matrix protein (Emdogain) in different time points.

There is clear evidence to support the routine use of conventional fluoride varnishes for the first step in the treatment of WSLs [6, 18, 19]. Fluoride application intends to harden the mineral surface layer, by its high affinity to hydroxyapatite, thus inhibiting the progression of the mineral loss [5, 7]. The fluoride ion does not enter into the subsurface layers if the appropriate fluoride concentration and frequency of application is not considered [20]. It has been verified that enamel demineralization around orthodontic brackets usually occurs at 30 days without any intervention [21]. In a recent split-mouth clinical study, Shah et al. [6] stated the first measurement time point as 45 days that found several changes in the demineralized enamel lesion depth. In addition, they concluded that Clinpro XT possibly provides a burst of fluoride released during the first several days. However, the possible time point remained uncertain. Therefore, a reasonable subsequent step was to initiate the first measurement at 7 days, as our samples were already demineralized. In the Clinpro XT group, fluorescence loss, lesion area, and volume were found to be significantly decreased at 21 days from the baseline. Kumar Jena et al. [22] evaluated the efficacy of Clinpro XT using DIAGNOdent and direct visual inspection and found significant reduction during orthodontic treatment. In another study [21], no demineralized enamel lesions were encountered up to 4 months in the Clinpro XT group. Shah et al. [6] also histologically found the absence of the demineralized lesion depth at an interval of 45, 90, and 120 days. However, the results of the abovementioned studies cannot be directly corroborating the present study since the reason was examining its therapeutic effect in resolving the artificially induced demineralized areas. Clinpro XT exhibits superior properties to conventional fluoride varnishes. It can be hypothesized that the fluoride stays in fluoroaluminosilicate glass particles: reaction at the surface provides the immediate release, while the interior provides a reservoir of fluoride for sustained release [6]. On the other hand, although the fluorescence loss and lesion area were decreased linearly, Duraphat did not significantly differ in enamel remineralization at 7, 14, and 21 days from the baseline. Since this result also supports previous researches [6, 23] that showed the low remineralization potential of the Duraphat varnish for the longer duration, conventional fluoride varnish can be recommended to be applied more frequently. The results of the present study also revealed that Enamel Pro Varnish had insufficient effects among the studied materials in treating the WSLs. Enamel Pro Varnish is another 5% sodium fluoride varnish that additionally contains amorphous calcium phosphate (ACP) formula [24]. These findings were attributed to the formation of ACP crystals and apatite on enamel surface.

The changes in the samples of the control group from baseline to day 21 suggest that some natural remineralization of WSLs occurred. Previous studies [23, 25] determined that the artificial saliva solution, which is frequently used as a negative control group in in vitro studies, displayed remineralizing potential for WSLs. Therefore, the decrease in fluorescence loss and lesion area can be contributed to the daily renewal of the artificial saliva at pH range between 6.5 and 6.8 throughout the whole present trial. However, it should be kept in mind that the remineralization was limited, and some specimens in the control group had no natural remineralization.

As expected, following the 14 days of the self-assembling peptide P_11-4_ (Curodont) application, a significant decrease was seen in Δ*F*, Δ*F* max, area, and Δ*Q* of the WSLs. The Curodont group showed higher QLF measurement changes between the first and second week in comparison to the Emdogain and Clinpro XT groups. Previous studies [8, 11, 23] comparing the remineralization activities of fluoride-containing agents also suggest Curodont as it represents the superior efficacy. In the two different in vitro studies [23, 26], self-assembling peptide P_11-4_ resulted a significant change in the fluorescence values at the 7th day, and an in vivo study carried out by Brunton et al. [27] showed similar findings of the remineralization in the lesion area from the 8th day. The mechanism of the self-assembling peptide P_11-4_ for the treatment of WSLs can be explained by the formation of de novo hydroxyapatite crystals. Since these structures are formed in a tangential form around the matrix fibers, a more “fan-type” structure is expected [7, 28]. However, the opacity of the natural enamel originates from the prismatic structure of the crystals; therefore, the entire fading of the WSLs cannot be expected following Curodont application.

Another notable result was that significant differences in fluorescence loss between baseline and the end of the observation period were found in samples on which Emdogain was applied. Although Emdogain, comprising enamel matrix proteins, has been used widely in dentistry [10, 11], up to date, there are a few studies [11–14] undertaken the direct application of Emdogain to evaluate the efficacy of remineralization stimulation potential of initial enamel caries. The results of the present study are quite similar to that of Schmidlin et al. [11] in which the application of enamel matrix proteins on artificial carious lesions seen to improve the re-hardening of the lesions, even in deeper layers. Together these findings suggest that Emdogain may promote remineralization of initial enamel carious lesions. This is consistent with a previous study [14] that confirmed the regeneration of the enamel prism-like tissue with the presence of Emdogain. Researchers attributed this effect to the molecular mechanisms of the product that include the prevention of the crystal fusion of premature crystals, the control of hydroxyapatite crystal morphology and elongation, and the control of the nucleation and regulation of the growth of the crystals in order to form the mineralized enamel covering the crowns of teeth [13, 14].

In this study, samples were acquired from bovine lower incisors in order to facilitate homogenous allocation of specimens from the same crown to the six test groups. Although human and bovine enamel are not identical in genetic and environmental features [29], it has been stated that bovine enamel could be in a better condition with a more uniform composition, particularly when a large crown size for preparing more samples from the same tooth was necessary [30]. Artificial caries lesions occurred rapidly in bovine enamel in comparison to the human teeth, even the mineral distributions of their lesions were almost equivalent [11, 31]. Since the results were only used for the interpretation of the comparison within the different groups of the present study and the changes within the same sample in different time points, we considered that the advantages of preferring bovine teeth will be acceptable.

The QLF-D is based on the same principle but with an enhanced light source and filter system compared with the traditional QLF method [32]. All specimens were dried and the images were captured with standardized settings. By using QLF-D, an in vitro standard was developed to capture the images in the same position and from the same angle. Unfortunately, the researcher in charge of product application knew which agent was applied. However, the examiners who conducted the imaging acquisition and the analysis of the images were blinded to the intervention allocation.

The results of this study revealed significant differences between initial and 21th days of QLF measurements in all groups except conventional fluoride varnishes. Although the present study did not mimic the exact real-life settings, the results have the potential to provide useful information on the remineralization effectiveness of different varnishes. Further in vivo studies would be beneficial to verify the treatment efficacy of different fluoride varnishes, enamel matrix protein, and self-assembling peptide on WSLs during or after orthodontic treatment.

## Conclusions

Within the limitations of this study, the following could be concluded:

- The null hypothesis was rejected. A significant regain in fluorescence was observed in all studied materials except the Duraphat and Enamel Pro fluoride varnishes. With respect to the reduction in lesion area, there were significant differences in the control, Curodont, and Clinpro XT groups at the end of the observation period.
- The fluorescence gain in the self-assembling peptide (Curodont) group was significantly higher compared to Emdogain and Clinpro XT between the first and second week of application. After 14 days, Clinpro XT varnish had the highest potential to decrease fluorescence loss and lesion area. It may be advisable to use combined of self-assembling peptides and light-curable fluoride varnish with prolonged duration of treatment period to increase the effect.

## Data Availability

The datasets used and/or analyzed during the current study are available from the corresponding author on reasonable request.

## Abbreviations

WSLs: White spot lesions
QLF: Quantitative light-induced fluorescence
Δ*F*: Fluorescence loss
Δ*Q*: Lesion volume
